# MaveQuest: a web resource for planning experimental tests of human variant effects

**DOI:** 10.1093/bioinformatics/btaa228

**Published:** 2020-04-06

**Authors:** Da Kuang, Jochen Weile, Roujia Li, Tom W Ouellette, Jarry A Barber, Frederick P Roth

**Affiliations:** b1 Donnelly Centre, University of Toronto, Toronto, ON M5S 3E1, Canada; b2 Department of Molecular Genetics, University of Toronto, Toronto, ON M5S 1A8, Canada; b3 Lunenfeld-Tanenbaum Research Institute, Sinai Health System, Toronto, ON M5G 1X5, Canada; b4 Department of Computer Science, University of Toronto, Toronto, ON M5T 3A1, Canada

## Abstract

**Summary:**

Fully realizing the promise of personalized medicine will require rapid and accurate classification of pathogenic human variation. Multiplexed assays of variant effect (MAVEs) can experimentally test nearly all possible variants in selected gene targets. Planning a MAVE study involves identifying target genes with clinical impact, and identifying scalable functional assays for that target. Here, we describe MaveQuest, a web-based resource enabling systematic variant effect mapping studies by identifying potential functional assays, disease phenotypes and clinical relevance for nearly all human protein-coding genes.

**Availability and implementation:**

MaveQuest service: https://mavequest.varianteffect.org/. MaveQuest source code: https://github.com/kvnkuang/mavequest-front-end/.

**Supplementary information:**

Supplementary data are available at *Bioinformatics* online.

## 1 Introduction

Driven by the advancement of genomic sequencing technologies, and by rapid increases in the number of identified disease-related genes and variants (Brunham and Hayden, 2013), clinical genetic testing is gaining increasingly broad use. An accompanying challenge is the frequent occurrence of (often extremely rare) variants that are difficult to interpret (Blazer *et al.*, 2015). In ClinVar, a popular resource for submitting genetic variants seen in clinical settings, approximately 40% of all variants are missense variants (Landrum *et al.*, 2016). Unfortunately, the majority of missense variants in ClinVar are now classified as ‘variants of uncertain significance’ (VUS) (Starita *et al.*, 2017; Weile and Roth, 2018), which makes any corresponding genetic tests not ‘clinically valid’ (Hoffman-Andrews, 2017), where clinical validity is defined by the extent to which a genetic test reveals a patient’s clinical phenotype or risk (Burke, 2014; Holtzman and Watson, 1999). Many purely computational methods, such as Polyphen-2 (Adzhubei *et al.*, 2010), have been established for predicting the functional effect of given variants. However, experimental functional assays can detect far more disease-associated variants with high confidence than can computational approaches (Sun *et al.*, 2016). Functional evidence is also considered important under the American College of Medical Genetics and Genomics/Association for Molecular Pathology guidelines (Richards *et al.*, 2015), and thus could help shift many VUS variants to more clinically useful categories (e.g. pathogenic or benign). However, conventional functional assays, such as complementation (Osborn and Miller, 2007), are often resource-intensive, and results from such assays are not generally available for rare clinical VUS variants.

Multiplexed assays of variant effect (MAVEs) provide a systematic, experimental approach to study nearly all missense variants in selected gene targets (Starita *et al.*, 2017). Indeed, some variant effect maps have been shown to outperform smaller-scale validated *in vitro* functional assays in quantitatively predicting disease phenotypes (Sun *et al.*, 2020).

The growing interest in MAVE studies (Weile and Roth, 2018) has presented bioinformatic challenges unique to the early planning stage. For example, to explore the clinical relevance of potential target genes and to identify scalable functional assays for these genes, information must be assembled from multiple database and literature resources. Here, we developed MaveQuest, a web-based service simplifying access to diverse aggregated information about potential functional assays, disease phenotypes and clinical relevance of genes for systematic variant effect mapping.

## 2 The database

**Fig. 1.**
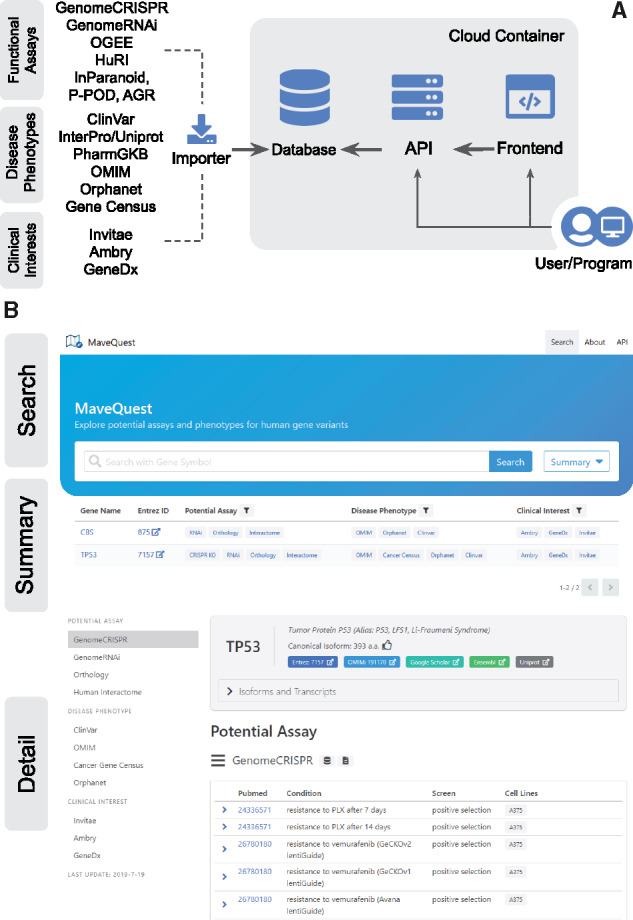
The architecture of MaveQuest. (**A**) Data from other sources were parsed and imported into the MaveQuest database and are retrieved by the API or the front-end user interface. (**B**) Three major components of the MaveQuest front-end service

The current version of the MaveQuest database curates literature for information related to 19 200 human genes from the Human Genome Organization’s Gene Nomenclature Committee collection (Braschi *et al.*, 2019). Of these genes, MaveQuest identified cellular phenotypes (each having the potential to enable a scalable functional assay) for 18 979 genes, disease phenotypes for 8460 genes and evidence of clinical relevance for 5203 genes. Figure 1A presents the three categories of data sources that were included in the MaveQuest database.

The first data category points the user to potential functional assays. GenomeCRISPR (Rauscher *et al.*, 2017), GenomeRNAi (Schmidt *et al.*, 2013) and the Online Gene Essentiality (Chen *et al.*, 2017) lead for human cell-based phenotypes that could form the basis of a scalable assay. The Human Reference Interactome Mapping project (Luck *et al.*, 2020) provides information on assays to identify variants that ablate specific protein interactions or generally reduce protein folding or stability. Data from InParanoid (Sonnhammer and Östlund, 2015), P-POD (Heinicke *et al.*, 2007) and Alliance of Genome Resources (Howe *et al.*, 2018) databases identify orthologs in non-human species, with links that allow the user to explore whether there are phenotypes associated with disruption of these orthologous genes that might be complemented by human genes to yield a scalable functional assay.

The second category provides data on disease phenotypes with which the query gene has been associated. ClinVar (Landrum *et al.*, 2018) provides clinically-interpreted variants reported for the query gene, which we can visualize to highlight regions enriched for pathogenic or benign variants, together with secondary structures, protein domains and families extracted from InterPro (Mitchell *et al.*, 2019) and Uniprot (UniProt Consortium, 2019) databases. To enable users to further evaluate the clinical significance of query genes, Online Mendelian Inheritance in Man (Hamosh *et al.*, 2005), Orphanet (INSERM, 1997), COSMIC Cancer Gene Census (Sondka *et al.*, 2018) and PharmGKB (Whirl-Carrillo *et al.*, 2012) databases summarize disease- and/or drug-related phenotypes, their mode of inheritance and, in some cases, molecular mechanisms.

The third category contains sequencing panels from three clinical genetic testing providers, Invitae, Ambry and GeneDx, who have each contributed many variant interpretations to ClinVar. The presence of a query gene in clinical genetic sequencing panels from multiple providers suggests clinical interest.

## 3 The application programming interface

The application programming interface (API) serves as an intermediate between the database and the front-end web application. The API, based on the RESTful standard (Richardson *et al.*, 2007), can be accessed directly using any common programming language. The API currently provides six functions (Supplementary Table S1) that could be further integrated with other MAVE resources as they emerge, e.g. MaveDB (Esposito *et al.*, 2019.)

## 4 The front-end web application

The front-end interface—enabling queries related to functional assays, disease phenotypes and clinical interests—contains three components (Fig. 1B). The first component, which also serves as the starting page, is a search panel that allows users to look up genes using identifiers. The second component is the gene summary page which lists cell-based phenotypes, disease phenotypes and evidence of clinical interest for each query gene. When the user has searched for partial matches, this information is included for all matching genes. Users can select a specific gene to bring up a detail page. This is the third component, which contains all data in the database associated with that gene. The detail page includes an overview of variants in ClinVar database when available, displaying the distribution of single-nucleotide variants along the protein sequence. Secondary structures, protein domains and families are also visualized for users to identify potential ‘variational hotspots’ (i.e. regions where variants are enriched). This feature is particularly useful for studying large proteins, allowing prioritization of regions harboring more pathogenic or benign variants.

## Supplementary Material

btaa228_Supplementary_Data
