# Supplementary material for: MaveQuest: a web resource for planning experimental tests of human variant effects
Source: Bioinformatics. 2020 Apr 6;36(12):3938–40. doi: 10.1093/bioinformatics/btaa228 (PMC7320626; doi:10.1093/bioinformatics/btaa228)
Supplement: btaa228_Supplementary_Data [file btaa228_supplementary_data.pdf]

Supplementary Table S1. A simplified summary of the application programming interface (API) of MaveQuest. A complete guide of the API service with sample requests and responses are available at https://mavequest.varianteffect.org/docs/api.

**Category**	**Method**	**Function**	**Description**
Summary	GET	/stats	Returns database statistics.
Summary	GET	/genes	Returns all records that passed a filter.
Gene	GET	/lookup	Returns primary ID and other information, given a gene in a certain species.
Gene	GET	/gene	Returns records, given gene(s) identifiers.
Gene	GET	/detail	Returns detailed records, given a gene symbol.
Interactome	GET	/interactome	Returns interaction details in HuRI database.
